# Clinical and humanistic impact of pharmacotherapeutic follow-up in patients with type 1 diabetes mellitus treated judicially

**DOI:** 10.1186/s13098-022-00835-8

**Published:** 2022-05-03

**Authors:** Thays S. Mendonça, William N. Oliveira, Vinícius S. Belo, Eduardo S. Silva, Mariana L. Pereira, Paulo R. Obreli-Neto, André O. Baldoni

**Affiliations:** 1 Programa de Pós-Graduação em Ciências da Saúde, Federal University of São João Del-Rei (UFSJ)–Campus Centro-Oeste Dona Lindu (CCO), Rua Sebastião Gonçalves Coelho, 400–Bairro Chanadour, Divinópolis, MG CEP:35501-296 Brazil; 2 Departamento de Farmácia, Centro Universitário das Faculdades Integradas de Ourinhos (UniFIO), Rodovia BR-153, Km 338 S/N Água do Cateto, Ourinhos, SP 19909-100 Brazil; 3 Núcleo de Ensino e Pesquisa em Farmácia Clínica (NEPeFaC), Federal University of São João Del-Rei (UFSJ), Rua Sebastião Gonçalves Coelho, 400 – Bairro Chanadour, Divinópolis, MG CEP:35501-296 Brazil

**Keywords:** Clinical pharmacy, Diabetes mellitus type 1, Insulin, Pharmaceutical care, Right to health

## Abstract

**Background:**

There is a lack of studies that assess the effectiveness of pharmacotherapeutic follow-up in the context of the judicialization of insulin analogues.

**Aims:**

To evaluate the clinical and humanistic impact of pharmacotherapeutic follow-up in patients with type 1 diabetes mellitus who receive insulin analogues by judicial decision in a Brazilian municipality.

**Methods:**

A quasi-experimental study of the before-and-after type was carried out through pharmacotherapeutic follow-up. Patients who accepted to participate in the study underwent laboratory tests of glycemic and lipid profile before and after the intervention, and underwent five pharmaceutical consultations. In addition, quality of life and health, knowledge, and skills related to insulin application techniques were analyzed.

**Results:**

28 patients participated in all stages. Of these, most were female (53.6%), with a mean age of 32.8 ± 11.6 years. After the intervention, there was a reduction in blood glucose levels, blood pressure, and increased body mass index. In addition, there was greater knowledge and skills regarding insulin application techniques, improved quality of life, health, greater number of medications used, reduction of pharmacotherapeutic problems, and improvement in eating habits.

**Conclusion:**

The pharmacotherapeutic follow-up promoted clinical and humanistic benefits, with improvement in quality of life and health.

**Supplementary Information:**

The online version contains supplementary material available at 10.1186/s13098-022-00835-8.

## Introduction

Diabetes mellitus type 1 (T1DM) accounts for 5–10% of diabetes mellitus (DM) cases worldwide. It is characterized as an autoimmune disease, caused by immune-mediated destruction of pancreatic beta cells [1]. As a result, the individual produces little or no insulin, and therefore there is a daily need to apply exogenous insulin to maintain blood glucose levels within normal limits [2]. In contrast, factors such as lack of adherence to treatment [3], associated with the complexity of pharmacotherapy, or even social and economic aspects, can directly affect the glycemic control of patients with DM [4, 5], and consequently, impact in increasing complications and mortality rates.

Evidence shows that the practice of effective care for patients with DM requires much more than heavy technology (medication); it requires a multidisciplinary team [6], and above all, collaborative relationships between professionals [7]. Hepler and Strand [8] established that the collaboration of the pharmacist with the health team in patient care takes place through the “responsible provision of medication therapy, with the purpose of achieving definitive results that improve the patient's quality of life” [8]. In this sense, the American Diabetes Association (ADA) recommends the incorporation of the pharmacist in the approach to the patient with DM, in the search for a more holistic treatment with better results [1].

From this perspective, the expansion of the role of pharmacists as members of health teams, can in fact produce better health outcomes for patients [9–11]. In recent years, pharmacotherapeutic follow-up services have made important contributions to individuals with chronic diseases and those using polypharmacy to prevent, identify, and solve pharmacotherapeutic problems (PP). Among the patients who benefit from this service are those with DM [12, 13], since pharmacotherapeutic follow-up can produce several clinical, economic and humanistic benefits, especially in relation to glycemic control [14–18], which in turn, reduces the progression of disease complications [19].

To our knowledge, there are incipient studies that have evaluated the effect of pharmacotherapeutic follow-up in patients with T1DM using insulin analogues via judicialization. Due to the high value of this pharmaceutical ingredient, lawsuits are frequently undertaken by patients with DM for the purchase of insulin analogues [21–23]. Between 2010 and 2014, insulin glargine was the most requested medication (6.3%), followed by insulin aspart (3.3%), among the lawsuits filed against the São Paulo State Department of Health [20]. Accordingly, between 2013 and 2017, insulin glargine (8%), lispro (3.5%), and aspart (2.6%) led the lawsuits filed against the state of Rio Grande do Sul [21].

Thus, investigations in this population scenario, in addition to enabling a better targeting of interventions, are necessary to improve the rationalization of finite health resources, and above all, to optimize the care of these patients. Futhermore, there are no studies that evaluate the clinical and humanistic impact of pharmacotherapeutic follow-up in patients with T1DM who acquire insulin analogues through the judicialization of health.

## Aim of the study

This study aims to evaluate the clinical and humanistic impact of pharmacotherapeutic follow-up in patients with T1DM who receive insulin analogues via judicialization.

## Methods

### Setting and participants

This is a quasi-experimental study of the before and after type, carried out through pharmacotherapeutic follow-up of patients who have T1DM and who receive insulin analogues by judicialization, in a medium-sized city in the state of Minas Gerais, Brazil. The study was based on criteria established by the checklist of the reporting of intervention evaluation studies using nonrandomized designs (TREND checklist). In the municipality there is a pharmacy for the exclusive dispensing of supplies and medications provided by judicialization, where the study was conducted during the period from April 2019 to August 2020 (see Additional file 1).

### Inclusion criteria

In 2019, there were 93 patients with T1DM who were receiving insulin analogues judicially. All were invited to participate in the study, with the exception of patients unable to attend the pharmacy for pharmaceutical consultations, such as bedridden patients and those who do not live in the municipality.

### Intervention

**Fig. 1:**
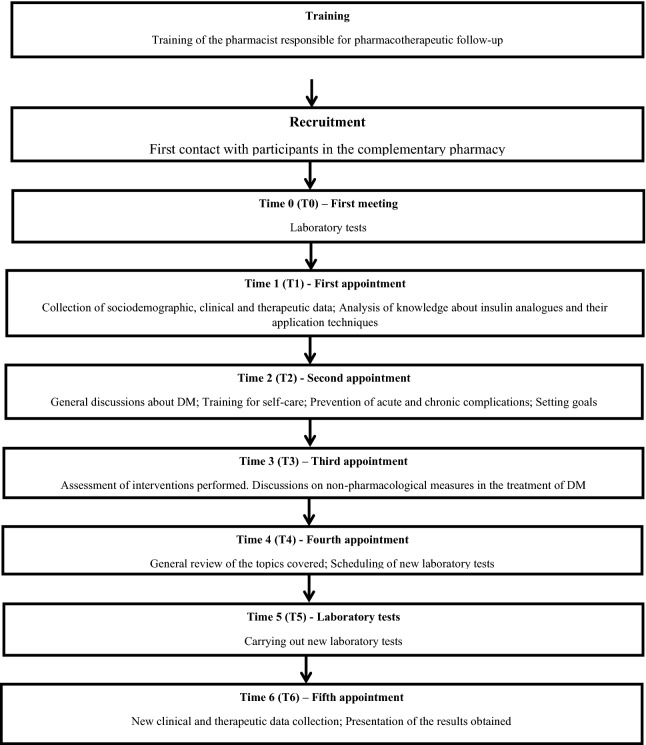
Flowchart representative of the study stages

The intervention (pharmacotherapeutic follow-up) was carried out in 9 stages (Fig. 1). Initially, the training of the pharmaceutical researcher responsible for the intervention was carried out, through training in theoretical and practical courses in pharmaceutical care. Subsequently, patients were recruited by the phamaceutical researcher. The study protocol was presented at the time they were looking for insulin analogues at the pharmacy. Patients who were registered at the pharmacy but who did not show up within the two-month recruitment period were contacted by telephone. Patients who agreed to participate in the research underwent tests in an outsourced clinical analysis laboratory, and underwent their first pharmaceutical consultation at the pharmacy's pharmaceutical office. In this consultation, questionnaires were applied to obtain sociodemographic, clinical, and therapeutic data, and regarding knowledge about insulin analogues and their skills in relation to application techniques. Participants went through three more pharmaceutical consultations (with 30-day intervals between them) and underwent laboratory tests again 30 days after the fourth consultation. In the fifth and in the last consultation, in turn, a new application of the questionnaires was carried out. Pharmaceutical consultations lasted an average of 20 to 40 min.

During the pharmaceutical consultations, aspects related to the general characteristics of DM and lifestyle modifications (LM) were discussed and presented in writing in booklet format: diet and exercise, pharmacological treatment (administration, storage, and disposal), monitoring of blood glucose, training for self-care, and prevention of acute and chronic complications, as proposed by the Brazilian Society of Diabetes (*Sociedade Brasileira de Diabetes*—SBD) (2020) [22]. Consultations were conducted following the method adapted from the Pharmacotherapy Workup (PW) [23] with the following stages: collection and organization of patient data, identification of pharmacotherapeutic problems (PP), elaboration of a care plan together with the patient, patient follow-up, and evaluation of the results of pharmaceutical interventions. According to the identification of the PP and based on the Clinical Protocols and Therapeutic Guidelines (CPTG), therapeutic goals and individual interventions were stipulated according to the needs of each patient. Due to the pandemic caused by the coronavirus Sars-Cov-2 (COVID-19), some patients had their last consultation remotely, as a means of prevention. In addition, three times a week, text messages were sent by cell phone applications to patients regarding topics covered in the consultations, in order to help them control the disease during the quarantine period.

### Variables and data source

Laboratory tests of fasting glucose, glycated hemoglobin (HbA1c), triglycerides, and total cholesterol and fractions were performed in an outsourced clinical analysis laboratory. The exams were performed before the first appointment and after the fourth appointment, with a 12-h fast. In all consultations, blood pressure (BP), waist circumference (WC), and weight were measured to calculate the body mass index (BMI).

An instrument was developed and validated to obtain sociodemographic, clinical, and therapeutic data, and on the classification of the patients' quality of life and health, which was applied in the first and last consultations for comparison. To obtain information about quality of life and health, patients were asked about the value they would give to their quality of life and health, which could range from 0 to 10. The *Data Collection Instrument –Group teaching of self-application of insulin at home* [24] was used to obtain information about the knowledge and skills of patients in relation to insulin application techniques. Thirteen questions were selected from this instrument, considering the correct content defined in the guidelines of the ADA (2020) and SBD (2017) [1, 25]. Each correct question scored 1 (one) point, with the result ranging from 0 to 13. The instrument was also applied in the first and last consultation.

During the consultations, educational materials such as sheets and booklets were given to help patients in self-care—Sheets: “Monitoring blood glucose levels”; “Medication administration schedules”; “Pharmacokinetic profile of insulins”; “Guidelines on diabetes” developed and validated by Aquino et al., (2016) [26]; and the prepared and validated booklets for this study: “Learn how to measure your capillary blood glucose”; “How to use insulin”; “Insulin pen: how to use” and “Insulin mixing technique” [27], which can be found on the website of the Academic League of Clinical Pharmacy (*Liga Acadêmica de Farmácia Clínica*—LAFarc) of the Federal University of São João del-Rei (UFSJ), (www. ufsj.edu.br/lafarc). In addition, personalized materials were delivered at each meeting as a way of minimizing the loss of patients in the study. The booklets and the instrument developed to obtain sociodemographic, clinical, and therapeutic data were validated through the Delphi technique. A questionnaire was created and was sent to 32 expert panelists in the area. In order to assess the consensus among the panelists' answers, in each item the calculation of the Content Validity Coefficient (CVC) was used, which varies from 0 to 1. A CVC above 0.8 was considered valid.

### Statistics

After descriptive analysis, laboratory and clinical parameters (fasting glucose, HbA1c, triglycerides, total cholesterol, LDL cholesterol, HDL cholesterol, VLDL cholesterol, systolic blood pressure (SBP), diastolic blood pressure (DBP), BMI and WC), therapeutic parameters (number of medications and PP), classification of quality of life and health (evaluated by values), knowledge and skills in the technique of insulin application (evaluated by the number of correct answers), and behavioral aspects related to non-pharmacological treatment (frequency of feeding, diet with restriction of sugars and carbohydrates, follow-up with a nutritionist and physical exercise) before and after the intervention were compared. Given the asymmetric distribution of data, assessed using the Shapiro test and normal quantile graphs, quantitative variables were compared using the Wilcoxon test. The differences of the means and the respective confidence intervals were calculated. Qualitative variables were compared using the McNemar test. Confidence intervals were calculated using the binomial exact calculation. For both tests, a significance level of 5% was adopted. Analyzes were conducted using the R program, version 4.0.3.

## Results

**Fig. 2:**
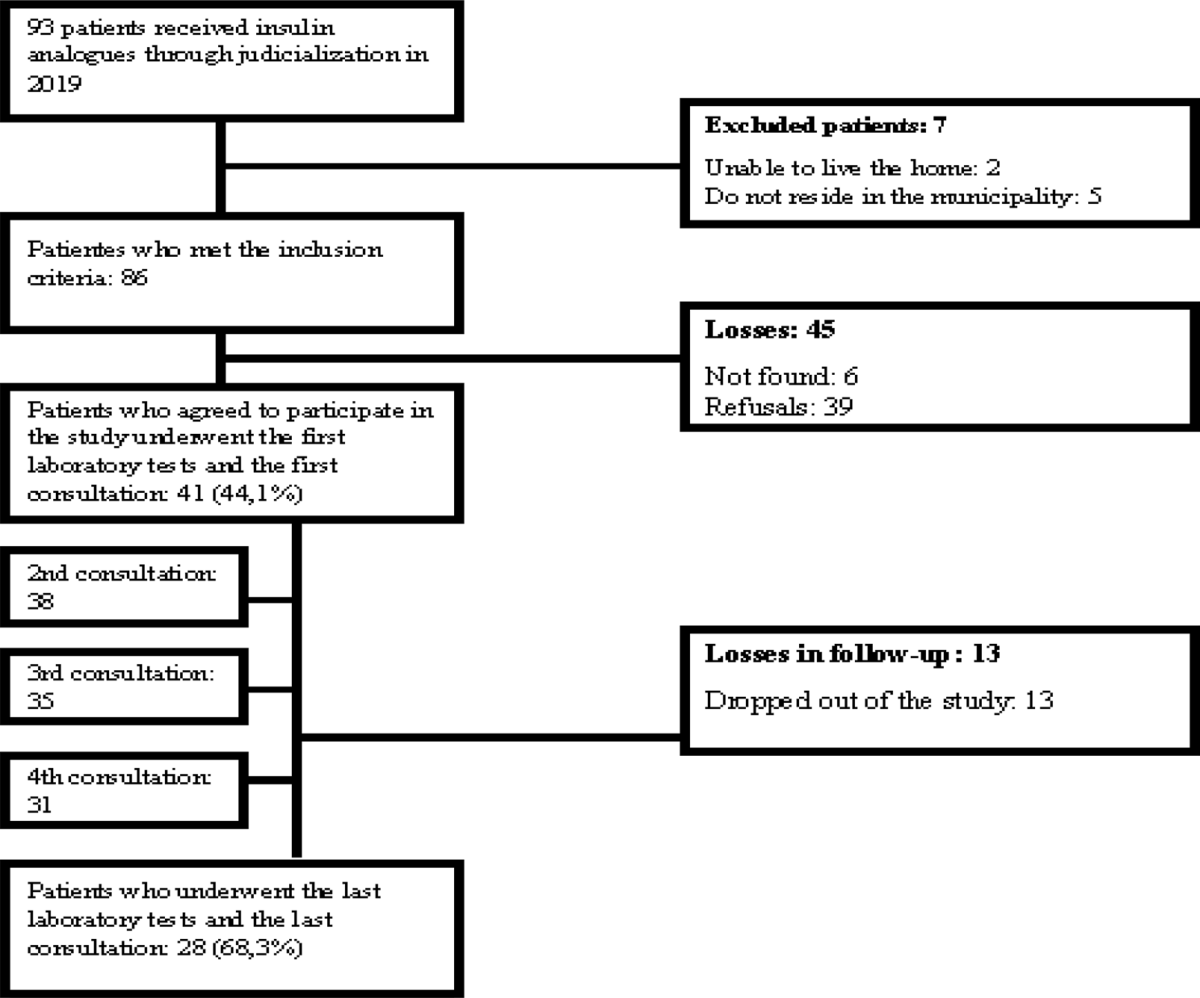
Patient recruitment and follow-up flowchart

Although all patients who met the inclusion criteria were invited to participate at the study, of the 93 patients who received insulin analogues through judicialization in 2019, 41 (44.1%) agreed to participate in the study. However, only 28 (68.3%) participated in all stages (Fig. 2). Most were female (n = 15; 53.6%), with a mean age of 32.8 ± 11.6 years. The mean time since diagnosis of T1DM for these participants was 20.2 ± 8.8 years and the mean time since the judicialization of insulin analogues was 9.0 ± 3.4 years.

In addition to T1DM, the most prevalent chronic diseases were dyslipidemia, systemic arterial hypertension (SAH) and hypothyroidism, present in 6 (21.4%), 5 (17.9%), and 5 (17.9%) patients respectively. The average number of chronic diseases per patient was 1.7 ± 0.9. Regarding the use of medications, it was identified that the participants used an average of 3.5 ± 1.8 medications. The majority used two insulin analogues (n = 23; 82.1%), the most frequent being the rapid acting insulin analogue aspart (89.3%) and the slow acting insulin analogue glargine (71.4%).

A total of 52 PP were identified in the first consultation and the vast majority were effective (n = 43; 82.7%). Mean PP per person at baseline was 1.9 ± 0.8 PP. After the intervention, it was possible to resolve 86.5% of the PP (n = 45) and an average of 0.3 ± 0.64 PP per person was obtained.

When comparing the laboratory and clinical parameters before and after the intervention, it was observed that after the pharmacotherapeutic follow-up, there was a significant reduction in the values of fasting glucose (p = 0.007), HbA1c (p = 0.005), and SBP (p = 0.001), in addition to an increase in BMI values (p = 0.004). The lipid profile showed improvement in its levels, with a reduction in triglycerides, total cholesterol, LDL cholesterol, VLDL cholesterol, and an increase in HDL cholesterol, but without statistical significance (Table 1).

**Table 1:** Comparison of laboratory and clinical parameters before and after the intervention (n = 28)

Variable	Before	After	Difference	IC	P value
Fasting blood glucose (mg/dL)	165.8 ± 74.8	120.8 ± 57.6	45.0	(10.03–79.97)	0.007*
HbA1c (%)	8.4 ± 1.8	7.9 ± 1.6	0.5	(0.39–1.39)	0.005*
Triglycerides (mg/dL)	95.9 ± 90.2	76.7 ± 36.2	19.2	(− 16.8–55.2)	0.380
Total cholesterol (mg/dL)	184.6 ± 56.4	174.0 ± 35.3	10.6	(− 14.04–35.24)	0.356
LDL cholesterol (mg/dL)	106.1 ± 43.7	97.1 ± 25.8	9.0	(− 9.80–27.80)	0.374
HDL cholesterol (mg/dL)	60.2 ± 15.6	60.6 ± 16.5	− 0.4	(− 8.81–8.01)	0.624
VLDL Cholesterol (mg/dL)	18.3 ± 10.3	16.5 ± 4.8	1.8	(− 2.41–6.01)	0.733
SBP (mmHg)	121.4 ± 26.5	110.4 ± 19.3	11.0	(− 1.14–23.14)	0.001*
DBP (mmHg)	72.9 ± 13.6	69.3 ± 12.1	3.6	(− 3.14–10.34)	0.140
BMI (Kg/m^2^)	23.7 ± 3.6	24.2 ± 3.5	− 0.5	(− 2.36–1.36)	0.004*
WC (cm)	85.3 ± 10.7	83.5 ± 9.9	1.8	(− 3.60–7.20)	0.051

*HbA1c* glycated hemoglobin, *SBP* systolic blood pressure, *DBP* diastolic blood pressure, *BMI* body mass index, *WC* waist circumference

*p value < 0.05 = statistical significance

After the intervention, there was an increase in the patients' knowledge and skills in relation to insulin application techniques (p < 0.001), as well as an improvement in their quality of life (p = 0.005) and their health (p = 0.018). Regarding therapeutic parameters, there was a reduction in the amount of PP (p < 0.001) and an increase in the amount of medication used by patients after pharmacotherapeutic follow-up (p = 0.046).

Regarding the behavioral aspects related to the non-pharmacological treatment of T1DM, there was an increase in the number of patients who started to eat every 3 h (p = 0.012) and to follow a diet with restriction of sugars and carbohydrates after the follow-up (p = 0.006). After the intervention, no statistical difference was observed between the number of patients who were followed by a nutritionist and who practiced physical exercise (Tables 2, 3).

**Table 2:** Comparison of parameters before and after the intervention, Brazil (n = 28)

Variable	Before	After	Difference	IC	P value
Number of medications	3.5 ± 1.8	4.1 ± 2.3	− 0.6	(− 1.68–0.48)	0.046*
PP number	1.9 ± 0.8	0.3 ± 0.6	1.6	(1.23–1.97)	< 0.001*
Quality of life (value from 0 to 10)	6.5 ± 2.0	7.5 ± 1.7	− 1.0	(− 1.97–0.02)	0.005*
Health (value from 0 to 10)	6.8 ± 2.1	7.5 ± 1.9	− 0.7	(− 1.75–0.35)	0.018*
Knowledge and skills (number of correct answers)	4.4 ± 2.0	10.2 ± 1.7	− 5.8	(− 6.77–− 4.83)	< 0.001*

*PP* pharmacotherapeutic problems

*p value < 0.05 = statistical significance

**Table 3:** Comparison of behavioral parameters related to non-pharmacological treatment before and after the intervention, Brazil (n = 28)

Variable	Before n (%)	IC	After n (%)	IC	P value
Eating frequency					
Every 3 h	14 (50)	(0.31–0.69)	23 (82.1)	(0.63–0.93)	0.012*
Every 4 h or more	14 (50)	(0.31–0.69)	5 (17.9)	(0.06–0.37)	
Diet with restriction of sugars and carbohydrates					
Yes	9 (32.1)	(0.16–0.52)	22 (78.6)	(0.59–0.92)	
No	8 (28.6)	(0.13–0.49)	3 (10.7)	(0.02–0.28)	0.006*
Partially	11 (39.3)	(0.22–0.59)	3 (10.7)	(0.02–0.28)	
Follow-up with a nutritionist					
Yes	6 (21.4)	(0.08–0.41)	4 (14.3)	(0.04–0.33)	0.500
No	22 (78.6)	(0.59–0.92)	24 (85.7)	(0.67–0.96)	
Physical exercise practice					
Yes	14 (50)	(0.31–0.69)	19 (67.9)	(0.48–0.84)	0.267
No	14 (50)	(0.31–0.69)	9 (32.1)	(0.16–0.52)	

^*^p value < 0.05 = statistical significance

## Discussion

After performing the pharmacotherapeutic follow-up, in the context of judicialization it was possible to observe a significant improvement in the glycemic levels of patients with T1DM, as demonstrated in studies carried out with patients with diabetes mellitus type 2 (T2DM) [12]. In the present study, the intervention provided a 0.5% reduction in the mean value of HbA1c. This result reinforces the importance of pharmacotherapeutic follow-up in patients with DM, since the control of HbA1c levels in these patients has an impact on the prevention of clinical complications. The literature explains that every 1% reduction in HbA1c reduces the risk of amputations by 43%, the risk of microvascular complications by 37%, and the risk of acute myocardial infarction by 14% [28].

As a consequence of the reduction in clinical complications, the decrease in HbA1c levels also has the potential to generate cost savings. As noted by Wagner et al. [29], a 1% reduction in HbA1c can save $685.00 to $950.00 per patient per year [29]. Following this logic, with the results obtained in the present study, savings of $342.50 to $475.0 per patient per year could be generated. It is important to consider that the cost of care related to DM corresponds to about two to three times more when compared to patients who do not have the disease [30] and that patients with poor glycemic control generate significantly higher expenses than patients with adequate blood glucose levels [31]. Additionally, special attention should be paid to the large number of lawsuits regarding the treatment of these patients, especially insulin analogues [32]. Thus, it can be said that the implementation of the pharmacotherapeutic follow-up service can contribute to the rationalization of the judicialization of health. It is clear that the pharmacotherapeutic follow-up in patients with T1DM from the moment of its diagnosis can contribute to the reduction of the judicialization of insulin analogues, since the proper use of insulin promotes greater control of pharmacotherapy.

In addition to glycemic control, pharmacotherapeutic follow-up significantly impacted SBP. However, the reduction in DBP was not statistically significant, similar to other studies analyzed [33]. A significant reduction in SBP was also observed in a study conducted by Santschi et al. [34]. In this meta-analysis comprising 39 randomized clinical trials, pharmaceutical interventions were associated with a reduction in SBP and DBP of − 7.6 mmHg (95% CI − 9.0; − 6.3) and − 3.9 mmHg (95% CI − 5.1; − 2.8), respectively [34]. According to Korcegez et al. (2017) these outcomes may have resulted from diabetes education, and above all, from the improvement of health behaviors, adherence, and pharmacotherapy management arising from pharmacotherapeutic follow-up [35].

After the intervention, a significant increase in BMI was observed, suggesting that patients with T1DM, in an attempt to optimize their insulin treatment, may experience weight gain. This can be explained by the fact that insulin participates in the regulation of lipogenesis and basal metabolism, in addition to inhibiting protein catabolism [36]. The risk of weight gain has been previously reported in patients with DM [37]. A study carried out in the United States in 2013 showed that young people with T1DM, followed for 24 months, had weight gain associated with the concomitant achievement of glycemic control, which can be partially explained by the increase in insulin administration [38]. In contrast, in a study conducted by Lipsky et al. [39], young people with T1DM followed for 18 months did not have BMI associated with glycemic control [39].

A 2011 prospective clinical trial conducted in DM patients in a city in the state of São Paulo showed that significant reductions in BMI (− 0.1 kg/m^2^; p < 0.001) and WC (− 0.6 cm; p < 0.001), over 36 months of follow-up, were observed in the group that received intervention from the clinical pharmacist, associated with reductions in the mean values of fasting glucose (− 27.2 mg/dL; p < 0.001) and HbA1c (− 0.7% p < 0.001). However, the study population was not exclusively composed of patients with T1DM, and thus the frequency of insulin use (10.3%) was lower than the other studies presented here [40].

Even in light of scientific evidence, we need to consider that the variability found in relation to weight gain can be attributed to different lengths of time in the follow-up and clinical characteristics of the study participants. Altogether, it is necessary to emphasize that in the present study, the adiposity measurement was based almost exclusively on the BMI and WC (with a non-significant reduction). In this sense, the association between improved glycemic control versus BMI can be explained differently, since it is an imprecise measure [41] that may not truly represent the distribution of body fat of the participants in this study.

In addition to the effects found in laboratory and clinical parameters, there was an increase in patients' knowledge and skills regarding insulin application techniques after the intervention. These data are consistent with those obtained in the study by Batista et al. [24], in which most patients did not have the knowledge and skills necessary for the correct use of insulin, which were obtained from the health education process [24]. Through these data one can see the importance of following-up the correct use of medications, since according to Flora and Gameiro [42], patients with T1DM have difficulties in administering insulin, especially with regard to the adjustment of doses [42]. Thus, obtaining knowledge and skills related to administration techniques can contribute to the reduction of PP related to these medications.

Following this premise, the findings of this study show the importance of pharmacists for the optimization of therapy, since pharmacotherapeutic follow-up significantly reduced the amount of PP. The results corroborate the findings of a 2018 cohort in Indonesia, in which the authors found that the incidences of PP in the management of T2DM with pharmaceutical intervention were significantly reduced compared to usual care [43]. A pharmaceutical intervention study conducted by Aquino et al. [12] showed a resolution of 60.9% of the baseline PP [12]; another study by Chung et al. [44] also found positive results regarding the reduction of PP after pharmaceutical intervention [44].

In the present study, the identification of gaps in the pharmacotherapy of patients, such as the untreated health condition, generated referrals to the prescriber, which may have driven the significant increase in the amount of medications (p = 0.046). A study carried out by Pepe et al. [45] demonstrated that 77% of the total pharmaceutical interventions were conducted to initiate the use of a new medication [45]. According to Houle et al. [46] new interventions have the potential to influence the increase in resource consumption and even raise costs [46]. Therefore, we believe that this result can be attributed to the consequent resoluteness of the intervened PP.

In addition to pharmacological treatment, pharmacotherapeutic follow-up also provided positive results in the non-pharmacological treatment of DM. According to Peres et al. [47] patients with T1DM often do not follow dietary and exercise recommendations. This leads to glycemic imbalances, which culminate in the need for complex therapeutic regimens [47]. A large number of participants in this study did not follow an adequate diet and did not practice physical exercise. After the pharmacotherapeutic follow-up, an improvement in feeding was observed, elucidating that it can contribute not only to the optimization of pharmacotherapy, but also to aspects of LM associated with the disease. A review study conducted with patients with DM found that changes in the patients' lifestyle had benefits for glycemic control in a similar way to the use of medications [48]. The lack of statistical significance for the increased practice of physical exercise can be explained by the occurrence of the pandemic generated by COVID-19, which triggered a period of social distancing with the closing and restriction of clubs, public spaces, and gymnasiums.

The previously discussed results corroborate the improvement in the quality of life and health of the participants. By promoting a reduction of blood glucose levels, SBP, improvements in diet, medication consumption (observed by the resolution of PP), and in knowledge and skills in relation to insulin application techniques, there is as a consequence, an improvement in health and quality of life. In this context, the importance of the clinical pharmacist in the care of patients with DM is highlighted. According to Coradi et al. [49] and Rahmathullah 50, pharmacotherapeutic follow-up provides improvements in blood glucose levels, adherence to pharmacotherapy, correct and rational use of medications, knowledge about the disease, as well as promoting a better quality of life for patients, and generates cost savings for health services [51].

Regarding the sociodemographic profile, most patients are female and young individuals. These data were as expected, as women are usually more concerned about their health than men, and T1DM affects younger individuals [1]. As demonstrated in previous studies referring to the judicilization of health, the insulin analogues aspart and glargine were the most used by the patients analyzed [20]. The most prevalent diseases associated with T1DM were dyslipidemia, SAH, and hypothyroidism. These results corroborate those obtained by Peres et al. [47] in a study conducted in patients with T1DM, in which the main diseases presented were SAH, dyslipidemia, and thyroid disorders, respectively, and by Coradi et al. [49] who found SAH and dyslipidemia as the main comorbidities in patients with T2DM [52].

The present work has some limitations. The number of participants was reduced due to patients' refusal, and loss of follow-up, which could lead to a selection bias, with a different profile of participants from those who did not accept to participate or who left in the middle of the study. This also made it impossible to carry out randomization and a control group. In addition, there is the possibility of an information bias due to the fact that data collection instruments depend on patients' self-reports. Additionally, the pandemic caused by COVID-19 did not allow the last consultation of all patients to be carried out in person. However, this is the first known study to assess the effectiveness of pharmacotherapeutic follow-up in T1DM patients receiving insulin analogues through the judicialization of health. With the results obtained, implementation strategies for pharmacotherapeutic follow-up can be structured within the scope of judicialization, with the aim of promoting benefits for patients, as well as generating cost reductions for the health system.

## Conclusions

The pharmacotherapeutic follow-up provided clinical and humanistic benefits, with improved quality of life and health of patients with T1DM who receive insulin analogues through judicialization. This shows that this type of service can be a great ally to ensure the promotion of the rational use of medications and the metabolic control of T1DM in patients, as well as contribute to minimizing the negative impacts of judicialization, especially the financial.

## Highlights

- The majority of the patients with type 1 diabetes mellitus (T1DM) who were attended by legal procedures do not have control of the disease.
- There was a clinical and humanistic improvement of these patients with pharmacotherapeutic follow-up.
- Pharmacotherapeutic follow-up can contribute to reduce the T1DM complications and promote better pharmacotherapy results.

## Supplementary Information

**Additional file 1:** Checklist of Transparent Reporting of Evaluations with Nonrandomized Designs (TREND).

## Data Availability

The datasets used and/or analyzed during the current study are available on request from the corresponding author.
